# Identification of immune‐enhanced molecular subtype associated with BRCA1 mutations, immune checkpoints and clinical outcome in ovarian carcinoma

**DOI:** 10.1111/jcmm.14830

**Published:** 2020-01-29

**Authors:** Mingjun Zheng, Yuexin Hu, Rui Gou, Ouxuan Liu, Xin Nie, Xiao Li, Qing Liu, Yingying Hao, Juanjuan Liu, Bei Lin

**Affiliations:** ^1^ Department of Gynaecology and Obstetrics Shengjing Hospital Affiliated to China Medical University Shenyang China; ^2^ Key Laboratory Of Maternal‐Fetal Medicine of Liaoning Province Key Laboratory of Obstetrics and Gynecology of Higher Education of Liaoning Province Shenyang China; ^3^ Department of Obstetrics and Gynecology University Hospital LMU Munich Munich Germany

**Keywords:** immune checkpoint, molecular subtypes, ovarian carcinoma, signatures, survival prognosis

## Abstract

Ovarian carcinoma has the highest mortality among the malignant tumours in gynaecology, and new treatment strategies are urgently needed to improve the clinical status of ovarian carcinoma patients. The Cancer Genome Atlas (TCGA) cohort were performed to explore the immune function of the internal environment of tumours and its clinical correlation with ovarian carcinoma. Finally, four molecular subtypes were obtained based on the global immune‐related genes. The correlation analysis and clinical characteristics showed that four subtypes were all significantly related to clinical stage; the immune scoring results indicated that most immune signatures were upregulated in C3 subtype, and the majority of tumour‐infiltrating immune cells were upregulated in both C3 and C4 subtypes. Compared with other subtypes, C3 subtype had a higher BRCA1 mutation, higher expression of immune checkpoints, and optimal survival prognosis. These findings of the immunological microenvironment in tumours may provide new ideas for developing immunotherapeutic strategies for ovarian carcinoma.

## 1 INTRODUCTION

Ovarian carcinoma (OV) is one of the most fatal gynaecological cancers.^1^ Although prognosis is improved to a certain degree by surgical treatment and platinum‐based chemotherapy, the majority of patients eventually die of recurrent tumour and platinum drug resistance, and the 5‐year survival of advanced patients is only 20%‐30%.^2^, ^3^ At present, ovarian carcinoma is mainly treated by surgery, chemotherapy, radiotherapy, targeted therapy, and endocrine therapy, but current therapies have a certain limited efficacy and fail to achieve satisfactory results. Therefore, more effective treatment measures are urgently required to improve the quality of life and survival of ovarian carcinoma patients. In recent years, tumour immunotherapy has attracted more and more attention, and it is a therapy that can eliminate cancer cells by enhancing the immune function of the human body.^4^ Compared with traditional tumour therapies, tumour immunotherapy mainly acts on the immune system or the microenvironment of tumours but not tumour cells, and it can also promote synergistic antitumour actions in combined treatment.^5^

Acquired immunity generally develops from the innate immune system and adaptive immune system and their interactions as well. The innate immune system produces immune cells (eg dendritic cells and macrophages) to protect the body, while the adaptive immune system defends against special threats via specific lymphocytes (B cells and T cells) to form immunological memory. Cancer cells disrupt the regulatory pathway of T cells, recruit immunosuppressive cells and release active cytokines with an immunosuppressive effect by influencing the antigen presentation process, thereby impairing the immune system and altering immune regulation for the benefit of the tumour cells.^6^, ^7^ By the advanced stage, they have developed several mechanisms to escape immune surveillance. The stimulation of programmed death‐1 (PD‐1) signal transduction in tumour cells facilitates the inhibition of T cell activity, and such inhibition can be promoted when the ligand CD86 or CD80 binds to CD28 or CTLA4. Thus, the upregulation of these immune checkpoint genes can lead to the suppression of the immune microenvironment.^8^ The immune checkpoint inhibitors developed for PD‐1 and CTLA4 can be effective in treating several tumours by activating the function of immune cells and normalizing the tumour microenvironment.^9^, ^10^ However, the efficacy of tumour immunotherapy is affected by the immune microenvironment of tumours, and some patients show significant response to tumour immunotherapy, so there is marked individual variation in the clinical treatment outcome.^11^ The complexity of the tumour immune microenvironment increases the difficulty of immunotherapy and affects its effectiveness, but the expression pattern of immune checkpoint genes in ovarian carcinoma and the potential clinical relationship are still unclear.^4^ Therefore, it is imperative to study in depth the overall immune status of patients, to identify the molecular subtypes of cancer, and to improve treatment efficacy in advanced OV patients.

This study aimed to explore the overall immune status of OV patients and its clinical significance. We screened the expression data of immune genes from the TCGA database and determined four molecular subtypes of ovarian carcinoma. We then compared the clinical characteristics, immune score, BRCA1/2 variant status, prognosis and immune checkpoint expression of the different subtypes, and finally validated our analysis results using external datasets. Our study findings can be helpful for the immunological treatment of ovarian carcinoma.

## 2 METHODS

### 2.1 Data source and processing

We used the GDC (https://docs.gdc.cancer.gov/API/Users_Guide/Getting_Started/) API to download the TCGA‐OV profile dataset containing a total of 374 samples and 5 samples of recurrent tumours, all of which were samples prior to standard treatment after diagnosis. Among them, Stage I, Stage II, Stage III and Stage IV have 1, 21, 292 and 57, respectively. We matched the expression profile and the clinical follow‐up samples and selected both samples as the sample set of the study. Further, we extracted the immune gene sets with expression from the expression profiles and selected the expression levels in each sample to be greater than 0. The sample with more than 30% of the genes was included as an immune gene for this study. Final inclusion of 1251 genes.

The GSE26193 dataset of the GPL570 platform was downloaded using the R package *GEOquery*, which contained 107 samples, of which Stage I, Stage II, Stage III and Stage IV were 21, 10, 59 and 17, respectively, according to the GPL570 annotation information. According to the annotation information of GPL570, probe mapping is applied to genes. If there are multiple probes corresponding to one gene, take the median and delete probes corresponding to multiple genes. Thirteen types of immune metagenes were collected from Safonov et al^12^ We downloaded 6 types of immune cells corresponding to each sample of OV from Timer (https://cistrome.shinyapps.io/timer/) and downloaded immune genes from ImmPort database (https://immport.niaid.nih.gov). We utilized the R package to estimate and calculate the immune score and matrix score of each sample.

### 2.2 Molecular subtypes screening based on immune genes

We made use of the expression profile of immune genes for consistent clustering, just as Zhang et al,^13^ who used R software package *ConsensusClusterPlus* to screen the molecular subtypes. In the study, Euclidean distance was utilized to calculate the similarity distance between samples, and K‐means was used for clustering. 80% of the samples were sampled by resampling scheme. Resampling was conducted for 100 times. The optimal number of clusters was determined by the cumulative distribution function (CDF). We further utilized the R package *sigclust* to analyse the clustering significance between these subtypes.

### 2.3 The relationship between subtypes and clinical features

Different clinical features are closely related to the development of the disease. The relationship between subtypes and disease development can be more clearly recognized by analysing the relationship between subtypes and clinical features. We extracted the information of age, grade and stage from the clinical follow‐up data of the patients and observed the relationship between the subtypes and age, grade, and stage, respectively.

### 2.4 The relationship between subtypes and immunity

There are key gene sets involved in the immune process discussed in previous studies. We collected 13 types of immune metagenes to analyse the relationship between these metagenes and subtypes. The immune components of tumour tissue are closely related to the prognosis of tumour. We analysed the relationship between matrix, immune score and molecular subtypes, respectively. The score of immune infiltrating cells directly reflects that the degree of immune infiltration in tumour tissue is closely related to the occurrence and development of tumour. We further utilized variance analysis to evaluate the differences in the above scores of different subtypes.

### 2.5 The relationship between subtypes and prognosis

We extracted the follow‐up data of patients from the sample follow‐up information and utilized K‐M to analyse the prognostic differences of different subtypes.

### 2.6 Other statistical methods

In this study, chi‐square test and exact test of Fisher's were utilized for the correlation between molecular subtypes and conventional clinical variables. The OS rates of all molecular subtypes were compared using log‐rank test and Kaplan‐Meier curves. All of the statistical tests were two‐sided tests. R software was utilized for statistical analysis.

## 3 RESULTS

### 3.1 Identification of four molecular subtypes of ovarian carcinoma based on immune profiles

**Figure 1:**
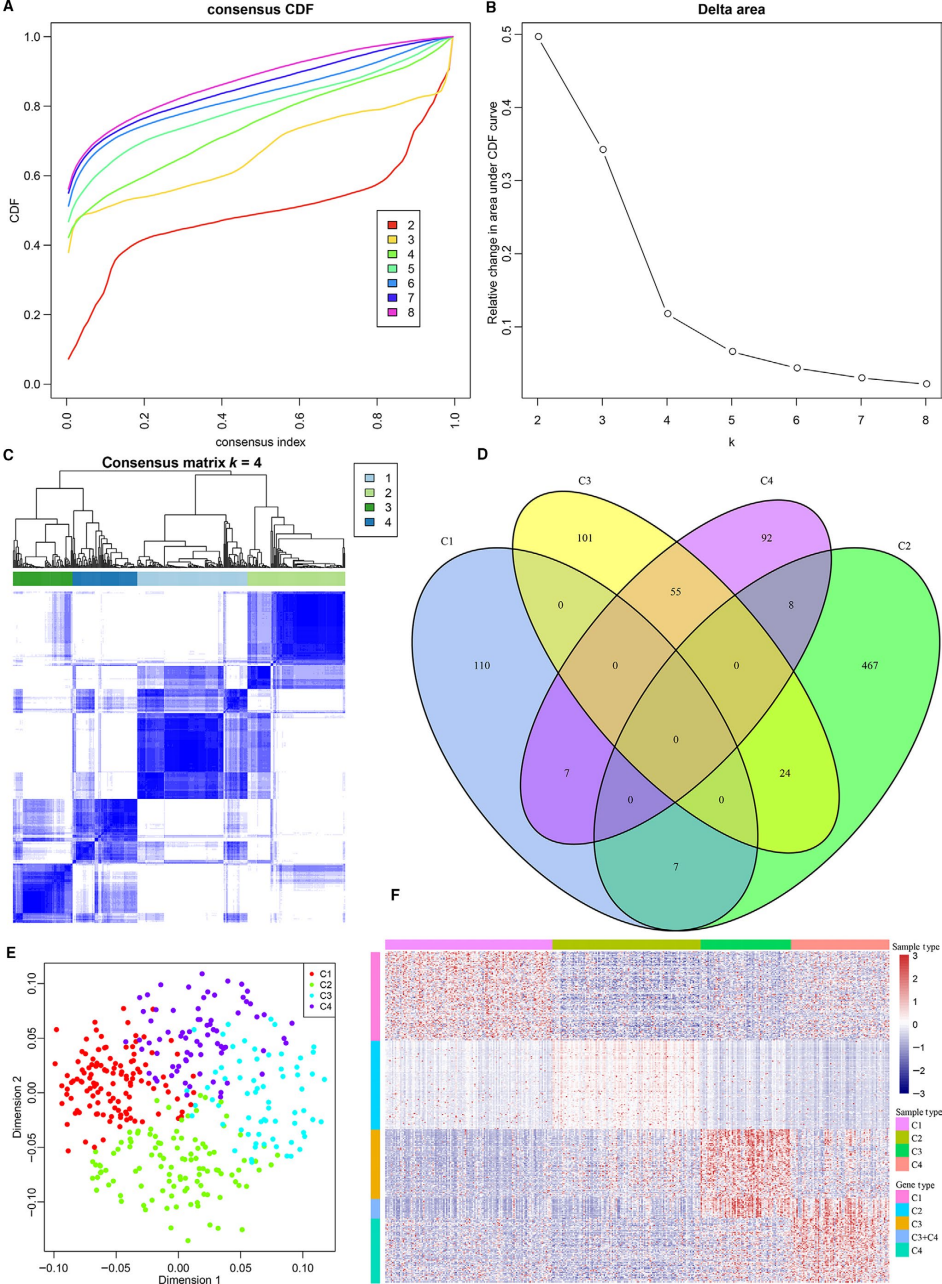
Identification of OV subtypes based on the immune genes. A, CDF curve; different colours reflect different cluster numbers, the horizontal axis represents the consensus index, the vertical axis stands for cumulative distribution function (CDF), and a bigger AUC indicates better clustering. B, CDF delta area curve of consensus clustering, indicating the relative change in area under the cumulative distribution function (CDF) curve for each category number *k* compared with *k* − 1. The horizontal axis represents the category number *k*, and the vertical axis represents the relative change in area under CDF curve. C, Heatmap of sample clustering at consensus *k* = 4; D, Intersection Venn diagram of significant high‐expression genes of various subtypes; E, Expression profile PCA of top 100 significant high‐expression genes, and scatter plot of top 2 components; F, gene expression heatmap of top 100 significant high‐expression genes in four subtypes. Red represents high expression, and blue represents low expression

The optimal number of clustering was determined by CDF. As shown in Figure 1A, the clustering results were stable when 4 subtypes were clustered, which were obtained by the subsequent observation of the CDF delta area curve in Figure 1B. Finally, *k* = 4 was selected and 4 molecular subtypes were obtained. The clustering significance among the 4 subtypes was further analysed by “*sigclust*” in the R software package, and the results indicated no significant clustering difference between C1 and C2 subtypes (*P* = 1) but a very significant clustering difference between C1 and C3, C1 and C4, C2 and C3, C2 and C4, and C3 and C4 subtypes (*P* = 0). The stable clustering results at *k* = 4 were chosen on the basis of the consensus clustering results. Figure 1C shows that 274 tumour samples were divided into these 4 subtypes. Furthermore, the expression spectra of 356 immune gene sets were used to analyse the differences between different subtypes, and the genes with a higher expression level in one subtype compared to other subtypes were screened using the Kolmogorov‐Smirnov test. Using FDR < 0.05 as the threshold, 124, 506, 180 and 162 genes with a higher expression level in C1, C2, C3 and C4, respectively, were eventually selected, and there was little intersection between these genes (Figure 1D). In addition, PCA was performed on the expression spectra of the top 100 genes with significantly higher expression for each subtype, and the scatter plot of the top 2 components is shown in Figure 1E, which indicates the clear clustering of the 4 subtypes. The expression profile heatmap of these genes is shown in Figure 1F, indicating that various subtypes had a clear border and a notable expression pattern in the expression spectra of these genes.

### 3.2 Relationship between 4 subtypes and clinical characteristics

The relationship between the 4 subtypes and age, tumour grade and tumour stage was analysed, as shown in Table 1. Four subtypes were not significantly correlated with age or grade but showed a significant relationship with stage, and Stage II samples of C3 subtype were evidently more than those of other subtypes.

**Table 1:** Relationship between 4 subtypes and clinical characteristics (*χ* ^2^ test)

	C1	C2	C3	C4	*P* value
Age					
>60	59	48	29	33	.9207
≤60	65	62	38	40	
Grade					
G1	0	0	0	1	.6647
G2	12	14	7	9	
G3	106	93	59	62	
G4	1	0	0	0	
Stage					
Stage I	1	0	0	0	.0257
Stage II	9	1	9	2	
Stage III	100	86	48	58	
Stage IV	14	22	9	12	

### 3.3 Relationship between 4 subtypes and immunity

**Figure 2:**
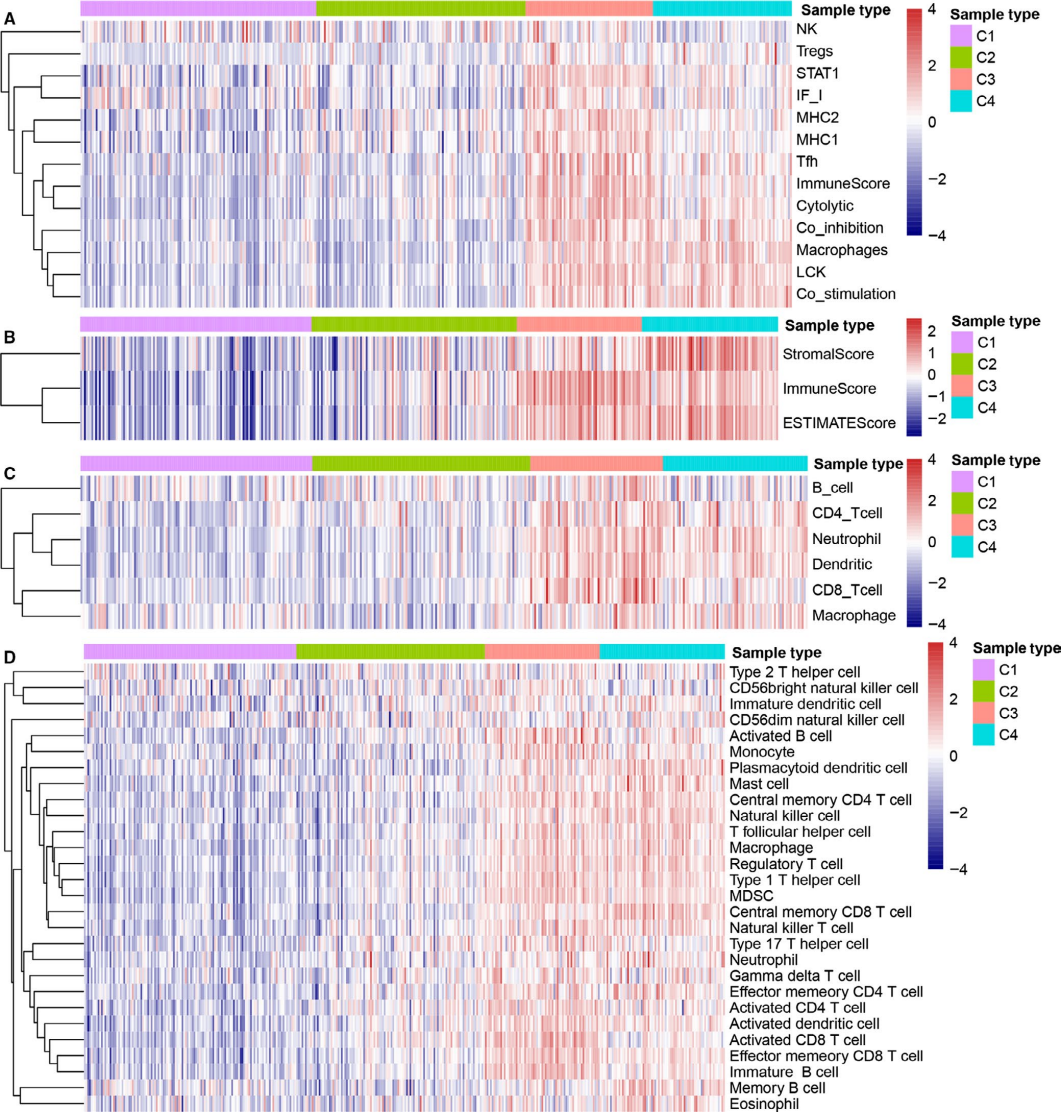
Immune profiles of the four molecular subtypes in the TCGA‐OV cohort. A, Gene expression score of 13 groups of immune metagenes in 4 molecular subtypes of ovarian cancer. In the heat map of gene expression, red represents high expression and blue represents low expression. B, Gene expression score of the tumour stroma scores, the immune scores and the tumour purity in 4 molecular subtypes of ovarian cancer. C, Gene expression score of 6 types of tumour‐infiltrating immune cells in 4 molecular subtypes of ovarian cancer. D, Gene expression score of 28 immune cell types in 4 molecular subtypes of ovarian cancer

To analyse the relationship between the 4 subtypes and immunity, we collected 13 immune metagenes,^12^ the scores of tumour immune components (matrix score, immune score and tumour purity) and the scores of 6 types of tumour‐infiltrating immune cells, and we then analysed the relationship between these three immunity‐related scores. The results showed that most of the 13 immune metagenes were highly expressed in C3, while a few were highly expressed in C3 and C4 (Figure 2A). The immunescore of C3 subtype was significantly higher than that of the other subtypes, and the matrix score and tumour purity of C4 subtype were clearly greater than those of the other subtypes (Figure 2B). Among 3 immune cell infiltration scores, B cell and CD8_cell scores of C3 were much higher than those of the other subtypes, and the scores of CD4_T cells, neutrophils, dendritic cells and macrophages in the C3 and C4 groups were markedly greater than those in the C1 and C2 groups(Figure 2C). Generally, most immune signatures in C3 subtype were upregulated as compared with C1 and C2 subtypes, and the upregulation of most tumour‐infiltrating immune cells was also observed in C3 and C4 subtypes, which indicated that the immune microenvironment of C3 and C4 subtypes was enhanced (Figure S1). Yang et al^14^ integrated a large number of ovarian cancer chip datasets, using gene set enrichment method to evaluate 28 kinds of immune cell types, which is more suitable in a variety of datasets. We used Yang et al's method to evaluate the enrichment score (ES) of samples in each subgroup among 28 immune cell types and found that most of them were highly expressed in C3. A few of them are highly expressed in C3 and C4, such as Figure 2D, which is consistent with our calculation of 13 immune metagene results (Figure 2A).

### 3.4 Analysis of prognostic differences and BRCA variant between 4 subtypes

**Figure 3:**
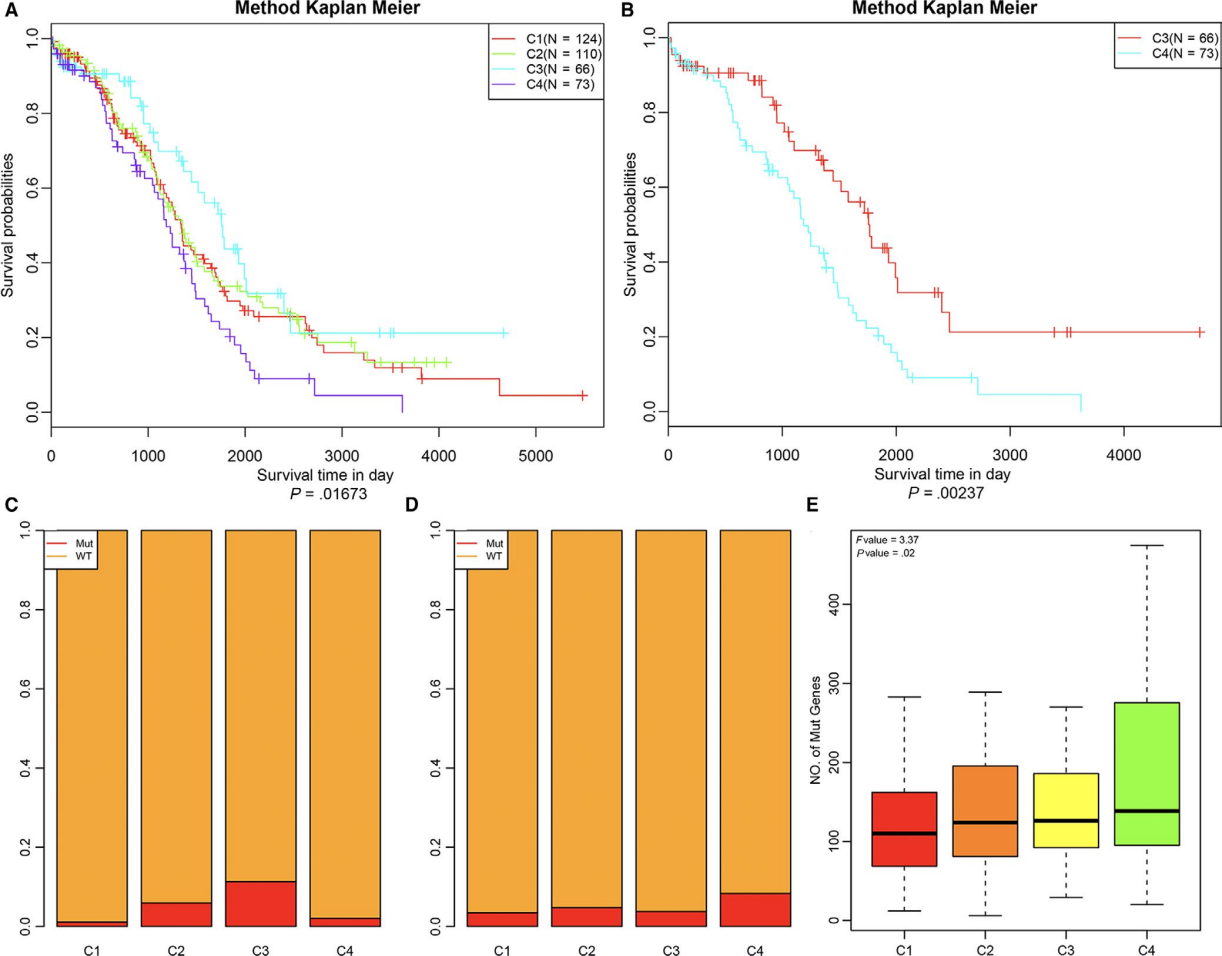
Survival analysis of the four ovarian cancer subtypes. A, KM curves showing prognostic relationship of 4 subtypes; The *P*‐value was calculated using the log‐rank test, by comparing the overall survival of 4 subtypes. The abscissa represents survival time (d) and the ordinate represents survival probabilities. B, KM curve showing prognostic difference between C3 and C4 subtypes. C, BRCA1 variant type/wild‐type ratio in 4 types of samples; D, BRCA2 variant type/wild‐type ratio in 4 types of samples; the abscissa from left to right is C1, C2, C3 and C4, and the ordinate represents ratio. E, Distribution of the number of mutated genes in 4 types of samples (ANOVA, *P* = .02). The abscissa from left to right is C1, C2, C3 and C4, and the ordinate represents number of mut genes

To determine the relationship between the 4 subtypes and prognosis, the prognostic differences between the 4 subtype samples were analysed by the Kaplan‐Meier method (Figure 3A). There was a significant difference in prognosis between the 4 subtype samples: the prognosis of C4 subtype samples was the worst, and the prognosis of C3 subtype samples was markedly better than that of the other subtype samples, while a very significant difference was found between C3 and C4 subtype samples (Figure 3B). This indicated that the immune‐enhanced subtypes C3 and C4 in ovarian carcinoma had two opposite clinical outcomes in the prognosis. BRCA is a tumour suppressor gene that plays an important role in the regulation of cell replication, the repair of DNA damage and the normal cell growth. BRCA variant loses tumour growth inhibiting function. There are up to 100 types of BRCA variant, and all BRCA variants are associated with human cancers, most closely with breast cancer followed by ovarian carcinoma. Therefore, we analysed the relationship of BRCA1 and BRCA2 variants in the 4 subtype samples. The data of BRCA1 and BRCA2 variants were extracted from SNP data in TCGA using MuTect, and the percentage of BRCA1 variant and non‐variant samples in the 4 subtype samples was then analysed separately (Figure 3C). The percentage of BRCA1 variant samples in C3 subtype samples was significantly higher than that in the other subtype samples (*χ* ^2^ test, *P* = .036). The BRCA2 variant/wild‐type ratio in the 4 subtype samples was analysed (Figure 3D), and it was greater in C4 subtype samples, but the differences with other subtype samples were not statistically significant (*χ* ^2^ test, *P* = .617). Furthermore, we analysed the distribution of the number of mutated genes in the 4 subtype samples and found a significant difference in the frequency of gene mutation between the 4 subtype samples; the frequency of gene mutation in C4 subtype samples was clearly higher than that in the other subtype samples (Figure 3E).

### 3.5 Relationship between 4 subtypes and the expression of 8 immune checkpoint genes

**Figure 4:**
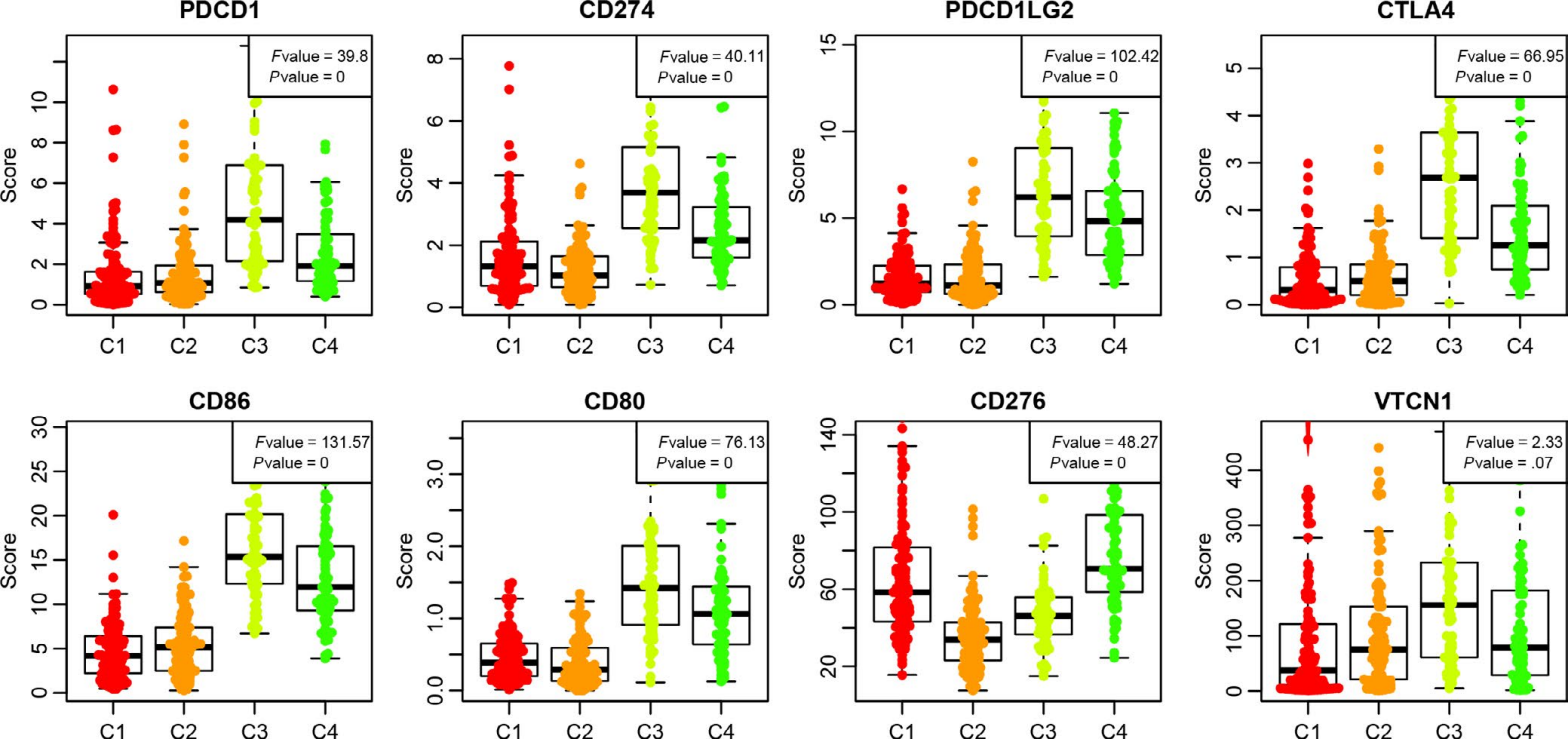
Validation of external datasets. A, Expression distribution of 13 immune metagenes in 4 subtypes in the validation set; B, Gene expression score of the tumour stroma scores, the immune scores and the tumour purity in the validation set; C, Expression distribution of 8 immune checkpoint genes in 4 subtypes in the validation set. The abscissa from left to right is C1, C2, C3 and C4, and the ordinate represents expression of 8 immune checkpoint genes (ANOVA); D, prognostic differences between 4 subtypes in the validation set; The P‐value was calculated using the log‐rank test, by comparing the overall survival of 4 subtypes. E, Prognostic difference between C3 and C4 subtypes. The abscissa represents survival time (d) and the ordinate represents survival probabilities

The relationship of 8 immune checkpoint genes with the 4 subtypes was further analyzed. The expression levels of PDCD1, CD274, PDCD1LG2, CTLA4, CD86 and CD80 in C3 subtype were significantly greater than those in other subtypes, and CD267 demonstrated a markedly higher expression level in C4 subtype (Figure 4). Relationship between 4 subtypes and the expression of 8 immune checkpoint genes. The abscissa from left to right is C1, C2, C3 and C4, and the ordinate represents expression of 8 immune checkpoint genes, comparisons of 4 subtypes were carried out by one‐way analysis of variance (ANOVA) test with post hoc contrasts by Student‐Newman‐Keuls test. The statistical significance for all tests was set at *P* < .05

### 3.6 WGCNA analysis and mining of immune‐enhanced subtype‐related modules

**Figure 5:**
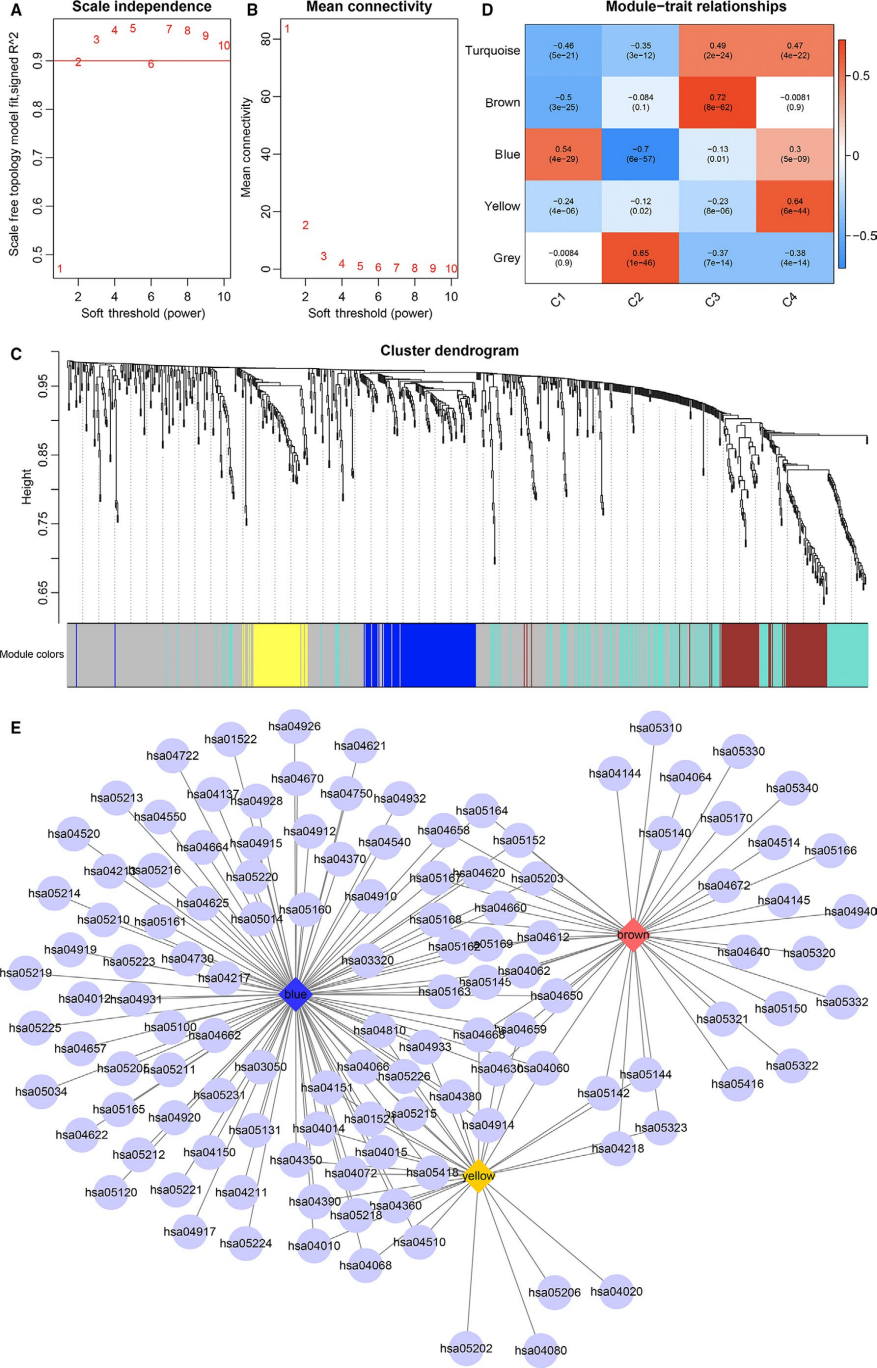
WGCNA analysis and mining of immune‐enhanced subtype‐related modules. A, Evaluation of the scale‐free model at different soft thresholds; a larger value indicates better compliance with the features of the biological network. B, Mean connectivity at different soft thresholds; the horizontal axis represents the soft threshold, and the vertical axis represents the mean connectivity analysis of network topology for various soft‐thresholding powers; C, Gene dendrogram and module colours; different colours represent the genes in different modules. D, Module‐feature correlation; the row represents the eigengenes of each module and the column represents the feature information of the samples. Red to green represents a high to low correlation coefficient. The digit in each grid indicates the correlation coefficient between gene modules and the corresponding features, and the digit in the bracket represents the *P* value. E, Enriched pathways associated with co‐expressed genes in blue module, yellow module and brown module. The diamond represents different modules, and the ellipse represents the path of enrichment

The expression profile data of 871 immune genes for the 4 subtypes were obtained to further mine the prognostic markers related to the immune microenvironment of ovarian carcinoma. The distance between transcripts was then calculated, and the weighted co‐expression network was constructed using WGCNA in the R software package.^15^, ^16^, ^17^, ^18^ Finally, the co‐expression modules were screened with the soft threshold of 2. The studies showed that the co‐expression network complied with the scale‐free network; that is, the log(*k*) of a node with the connectivity being *k* was negatively correlated with the log(*P*(*k*)) of the presentation probability of the node, and the correlation coefficient was >0.8. To make sure that the network was a scale‐free one, *β* = 2 was selected (Figure 5A,B). The expression matrix was converted into the adjacency matrix, and the latter was then converted into the topological matrix. The genes were clustered on the basis of TOM using the average‐linkage hierarchical clustering method, where the standard of three was sheared according to the mixed dynamics, and the minimum number of genes in each gene (lncRNA) network module was set at 30. After the determination of gene modules with the dynamic shear method, the eigengene of modules was calculated in sequence, and the modules were then subjected to clustering analysis, The adjacent modules were merged into the new modules, and the setting of height = 0.25, deepSplit = 2 and minModuleSize = 30 was done. Totally, 5 modules were obtained (Figure 5C), and it should be noted that the grey module could not be clustered into the gene sets of other modules. The transcript statistics of various modules are shown in Table S1, and 458 transcripts were divided into 4 co‐expression modules. The eigengenes of 5 modules and their correlations with the 4 subtypes were determined separately (Figure 5D); the blue module correlated positively with C1 but negatively with C2, the brown module and yellow module correlated with C3 and C4, respectively (average correlation coefficient > 0.65). The number of transcripts in the 3 modules was 115, 100 and 61, respectively, and a total of 276 genes were included. The functions of genes in the modules related to the 4 subtypes were further analysed, and KEGG enrichment analysis was conducted using *clusterProfiler* in the R software package at a significance level of FDR < 0.05. Among three modules, there were 42 pathways in the brown module (Table S2), 30 pathways in the yellow module (Table S3), 94 pathways enriched in the blue module (Table S4) (Figure 5E). The relationship of pathways enriched in these three modules was analysed, and a total of 121 pathways were enriched in three modules, where the pathways in the yellow modules overlapped mostly with those in the other two modules.

### 3.7 Validation of external datasets

**Figure 6:**
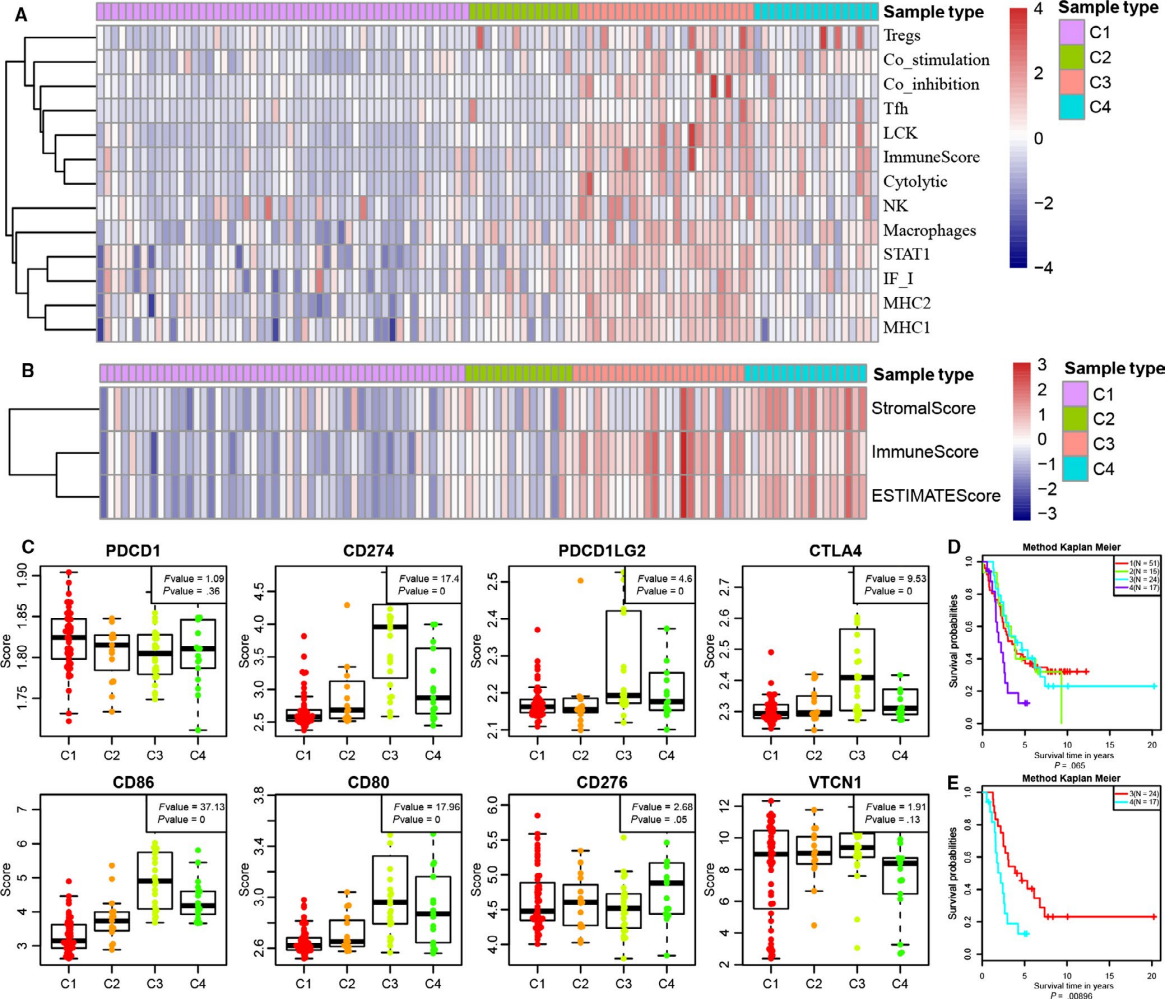
Validation of external datasets. A, Expression distribution of 13 immune metagenes in 4 subtypes in the validation set; B, Gene expression score of the tumour stroma scores, the immune scores and the tumour purity in the validation set; C, Expression distribution of 8 immune checkpoint genes in 4 subtypes in the validation set. The abscissa from left to right is C1, C2, C3 and C4, and the ordinate represents expression of 8 immune checkpoint genes (ANOVA); D, prognostic differences between 4 subtypes in the validation set; The *P*‐value was calculated using the log‐rank test, by comparing the overall survival of 4 subtypes. E, Prognostic difference between C3 and C4 subtypes. The abscissa represents survival time (d) and the ordinate represents survival probabilities

We selected the genes in the gene co‐expression modules (blue, brown and yellow) closely related to various subtypes and then extracted the expression spectra as a training set. The classification model was established using support vector machine (SVM), and the samples were then classified with an accuracy of 100%. To further validate the 4 subtypes, GSE26193 standard data including a total of 107 samples were downloaded from the GEO database, and thereafter, the expression spectra of genes in the blue, brown and yellow modules were extracted and substituted into the model for sample classification. There were 51 samples of C1 subtype, 15 samples of C2 subtype, 24 samples of C3 subtype and 17 samples of C4 subtype predicted. We first analysed the expression distribution of 13 immune metagenes in the 4 subtypes (Figure 6A) and found a high expression of most immune metagenes in C3 subtype, which was consistent with the training set. Next, we further analysed the immune scores of the samples (Figure 6B) and observed that the immune score in C3 subtype was significantly higher than that in the other subtypes and that the matrix score and tumour purity in C4 subtype were clearly greater than that in the other subtypes, which was coincident with the training set. The analysis of the expression distribution of 8 immune checkpoint genes is shown in Figure 6C, and 6 of 8 genes demonstrated an expression distribution consistent with the training set. On the basis of the analysis of prognostic differences (Figure 6D), the differences in prognosis between 4 subtypes were marginally significant (*P* = .065), and the prognosis of C4 subtype was markedly poorer than that of the other subtypes; as shown by further analysis, the prognosis of C3 subtype was clearly better than that of C4 subtype (Figure 6E), which was consistent with the validation dataset.

### 3.8 Data analysis flow chart

**Figure 7:**
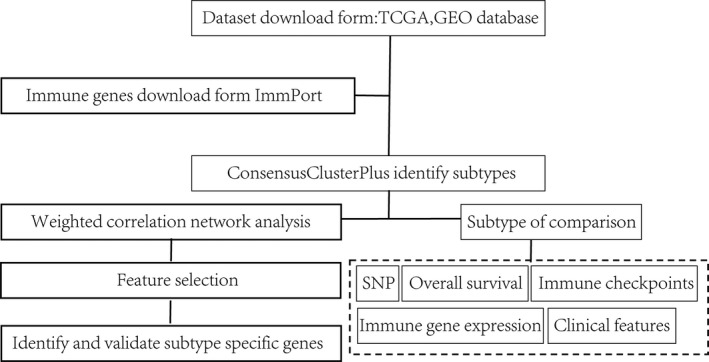
The workflow of the proposed method

To make our study better understand. The workflow of the proposed method was developed as shown in Figure 7.

## 4 DISCUSSION

In recent years, an increasing number of studies have focused on exploring the molecular subtypes of epithelial ovarian cancer based on genomewide profiles or multi‐omics to promote the realization of personalized treatment and improve the survival rate in patient^19^, ^20^, ^21^, ^22^; however, the achievements of molecular subtypes remain in the initial phase.

The immune process plays a key role in the carcinogenesis and progression of solid tumours. It is believed that the newly nascent transformed cells can be initially eliminated by the host immune system based on innate immunity and adaptive immunity, and the destroyed cells then release various tumour antigens, further stimulate adaptive immunity and activate T/B lymphocytes. Studies have shown that tumour lymphatic infiltration and better survivability of patients have a strong correlation,^23^, ^24^, ^25^ suggesting that patients with ovarian cancer may benefit from immunotherapy. Therefore, screening for immune molecular subtype based on ovarian cancer is of great clinical significance.

The advantage of current study mainly aimed to investigate the global immune profiles, which could provide more details about the immune landscape in ovarian cancer. We identified four gene expression subtypes based on the global immune genes in the TCGA‐OV cohort. It is verified in the external dataset GSE26193.

Among the four molecular subtypes, the overall immune profiles of subtypes C3 and C4 were significantly higher than in comparison with that of subtypes C1 and C2. However, there were also differences between C3 and C4. Most of the 13 kinds of immune metagenes and immune cell infiltration score (immunity) were highly expressed in C3 subtype (Figure 3A,B), and the immune cell infiltration score (matrix and tumour purity) of C4 subtype was significantly higher than those of other subtypes (Figure 3B). The scores of multiple types of immune‐related cells such as B_cell and CD8_cell in C3 subtype were significantly higher than those in other subtypes.

These findings indicate that the immune microenvironment of C3 and C4 subtypes was strengthened. C1 and C2 subtypes had lower immunoreactive expression with lower immune scores. In the comparison of overall survival, the prognosis of C1 and C2 subtypes was poor and consistent. It was noted that the poorest prognosis was found in C4 subtype, while C3 subtype had significantly better prognosis than the other subtypes. This suggests that the immune‐enhanced subtypes may not respond to the best prognosis in ovarian carcinoma.

BRCA1/2 is a tumour suppressor gene and encodes BRCA1/2 protein, which plays a critical role in regulating essential cellular activities, such as normal cell growth, repair, transcription and activation of DNA damage, and inhibition of chromatin remodeling.^26^ Mutated BRCA1/2 gene loses its normal physiological functions, so that the wrong non‐homologous recombination occurs in cells, resulting in the progression of cancer.^27^ Our analysis on the correlations between the 4 subtypes and BRCA1/2 mutation showed that BRCA1 mutation percentage in C3 subtype samples was significantly higher than that in the other subtype samples. Some studies have demonstrated that pathways involving BRCA1/2 play a very important role in the process of cisplatin resistance; the nucleotide excision repair pathway during DNA repair is little associated with cisplatin resistance, but such drug resistance could be enhanced by the upregulation of the homologous recombination repair pathway.^28^ BRCA1/2 directly or indirectly participates in DNA excision and repair. Meanwhile, BRCA1/2 absence or mutation can increase the sensitivity of cells to DNA crosslinkers (eg cisplatin), and the capacity of cisplatin drugs to damage DNA double strands will be enhanced if BRCA protein activity is lost.^29^ It has been shown that the 5‐year survival of patients with BRCA1 mutation is increased with cisplatin and paclitaxel combination chemotherapy,^30^ which may explain why C3 subtype with the highest BRCA1 mutation rate had a good prognosis in our study.

Recent studies have suggested that immune checkpoints play an important role in the immune escape of cancer,^31^ so we further analysed the relationship between the 4 subtypes and 8 immune checkpoint genes (PDCD1, CD274, PDCD1LG2, CTLA4, CD86, CD80 and CD267). These genes mainly encode high‐interest therapeutic targets, including PD‐1, programmed death‐ligand 1, programmed death‐ligand 2, cytotoxic T lymphocyte‐associated antigen‐4 (CTLA‐4), CD86, CD80 and 13b protein (a member of tumour necrosis factor receptor superfamily). Currently, the monoclonal antibodies developed for PD‐1 and its ligands are successfully applied in clinical practice and approved for several cancers (eg melanoma, non‐small cell lung carcinoma, renal cancer, and bladder cancer).^32^ Clinical studies have shown that the combination of anti‐PD‐1/PD‐L1 antibody and CTLA‐4 inhibitor can improve the treatment effect of patients with advanced melanoma, and it has been approved by the FDA for treating BRAF V600E wild‐type patients with unresectable or metastatic melanoma.^9^, ^10^ A recent phase‐II clinical trial (CheckMate 069) revealed that compared with monotherapy with ipilimumab (a CTLA‐4 inhibitor), the combination therapy of ipilimumab and nivolumab (a PD‐1 inhibitor) improved therapeutic efficacy in patients with advanced melanoma, and the 2‐year survival of monotherapy and combination therapy was 53.6% and 63.8%, respectively.^33^ Our study results suggested that PDCD1, CD274, PDCD1LG2, CTLA4, CD86 and CD80 were highly expressed in the samples of the C3 immune‐enhanced subtype. Many studies have confirmed that some immunosuppressive molecules (eg PD‐L1) are overexpressed when tumours invade the lymph nodes, and this process is called “adaptive immune resistance.” Therefore, the high expression of immunosuppressive molecules such as PD‐L1 and CD274 is not only the result of the mutations of tumour cells, but also possibly induced during immune cell invasion of tumours. Such high expression in the microenvironment of tumour lesions means a strong tumour immune attack, which is the reason for good prognosis in the patients with C3 immune‐enhanced subtype characteristics.

Next, we used WGCNA to explore the functions involved in genes in the four molecular subtypes of the ovarian cancer immune microenvironment. The results showed that the C3 subtype was significantly positively correlated with the brown module (*r* = .72, *P* = 8e−62), while the gene of the brown module was mainly enriched in the Antigen processing and presentation signal pathway (Table S2). The role of these pathways is closely related to malignant neoplasms, autoimmune reaction and inflammation. And the C4 subtype is also significantly positively correlated with the yellow module (*r* = .64, *P* = 6e−44). The related genes are significantly enriched in MAPK signalling pathway and PI3K‐Akt signalling pathway (Table S3); The blue module gene is significantly enriched in Axon guidance and EGFR tyrosine kinase inhibitor resistance signalling pathway (Table S4).

The results of the validation set showed that the overall immune profiles of subtypes C3 and C4 were significantly higher than in comparison with that of subtypes C1 and C2, and most of the immune checkpoint genes were highly expressed in the C3 and C4 subtypes, consistent with the training set. The reliability of the C3/C4 subtype as an enhanced subtype of the ovarian cancer immune microenvironment was further demonstrated.

## 5 CONCLUSION

From the above discussion, the conclusion can be reached that we identified two immune‐enhanced subtypes using gene expression profiles of global immune genes through large databases of TCGA and GEO. The two subtypes are distinct in immune checkpoint molecules, immune function, BRCA mutation and clinical prognosis. These findings of the immune microenvironment may shed new light on the strategy of immunotherapy in ovarian cancer. With the development of internet and big data era coming, constructing databases^34^, ^35^, ^36^ and establishing powerful webserver^37^, ^38^ will provide the convenience to most scholars.

## CONFLICT OF INTEREST

No potential conflict of interest to disclose.

## AUTHOR CONTRIBUTIONS

Mingjun Zheng, Yuexin Hu and Rui Gou conceived the idea for the paper. All authors read and approved the final manuscript.

## Data Availability

All data generated or analysed during this study are included in this article.

## References

[1] Banks E . The epidemiology of ovarian cancer. Methods Mol Med. 2001;39(6):3. 21340753 10.1385/1-59259-071-3:3

[2] Jayson GC , Kohn EC , Kitchener HC , Ledermann JA . Ovarian cancer. Lancet. 2014;384(9951):1376‐1388. 24767708 10.1016/S0140-6736(13)62146-7

[3] NCI . SEER Cancer Statistics Review, 1975‐2014. 2017.

[4] Quail DF , Joyce JA . Microenvironmental regulation of tumor progression and metastasis. Nat Med. 2013;19(11):1423–1437. 24202395 10.1038/nm.3394 PMC3954707

[5] Li Y , Li F , Jiang F , et al. A mini‐review for cancer immunotherapy: molecular understanding of PD‐1/PD‐L1 pathway & translational blockade of immune checkpoints. Int J Mol Sci. 2016;17(7):1151. 10.3390/ijms17071151 PMC4964524 27438833

[6] Antonia SJ , Larkin J , Ascierto PA . Immuno‐oncologycombinations: Are view of clinical experience and future prospects. Clin Cancer Res. 2014;20(24):6258‐6268. 25341541 10.1158/1078-0432.CCR-14-1457

[7] Finn OJ . Immuno‐oncology: understanding the function and dysfunction of the immune system in cancer. Ann Oncol. 2012;23(suppl 8):viii6‐viii9. 22918931 10.1093/annonc/mds256 PMC4085883

[8] Cao B , Wang Q , Zhang H , Zhu G , Lang J . Two immune‐enhanced molecular subtypes differ in inflammation, checkpoint signaling and outcome of advanced head and neck squamous cell carcinoma. Oncoimmunology. 2017;7(2):e1392427. 29308323 10.1080/2162402X.2017.1392427 PMC5749623

[9] Boutros C , Tarhini A , Routier E , et al. Safety profiles of anti‐CTLA‐4 and anti‐PD‐1 antibodies alone and in combination. Nat Rev Clin Oncol. 2016;13(8):473‐486. 27141885 10.1038/nrclinonc.2016.58

[10] Baumeister SH , Freeman GJ , Dranoff G , Sharpe AH . Coinhibitory pathways in immunotherapy for cancer. Annu Rev Immunol. 2016;34:539‐573. 26927206 10.1146/annurev-immunol-032414-112049

[11] Beatty GL , Gladney WL . Immune escape mechanisms as a guide for cancer immunotherapy. Clin Cancer Res. 2015;21(4):687‐692. 25501578 10.1158/1078-0432.CCR-14-1860 PMC4334715

[12] Safonov A , Jiang T , Bianchini G , et al. Immune gene expression is associated with genomic aberrations in breast cancer. Can Res. 2017;77:3317‐3324. 10.1158/0008-5472.CAN-16-3478 28428277

[13] Zhang S , Wang Y , Gu Y , et al. Specific breast cancer prognosis‐subtype distinctions based on DNA methylation patterns. Mol Oncol. 2018;12(7):1047‐1060. 29675884 10.1002/1878-0261.12309 PMC6026876

[14] Yang L , Wang S , Zhang QI , et al. Clinical significance of the immune microenvironment in ovarian cancer patients. Mol Omics. 2018;14(5):341‐351. 30129640 10.1039/c8mo00128f

[15] Hughes DA , Kircher M , He Z , et al. Evaluating intra‐ and inter‐individual variation in the human placental transcriptome. Genome Biol. 2015;16:54. 25887593 10.1186/s13059-015-0627-z PMC4404591

[16] Zuo Y , Su G , Wang S , et al. Exploring timing activation of functional pathway based on differential co‐expression analysis in preimplantation embryogenesis. Oncotarget. 2016;7(45):74120‐74131. 27705919 10.18632/oncotarget.12339 PMC5342040

[17] Bakken TE , Miller JA , Luo R , et al. Spatiotemporal dynamics of the postnatal developing primate brain transcriptome. Hum Mol Genet. 2015;24(15):4327‐4339. 25954031 10.1093/hmg/ddv166 PMC4492396

[18] Zuo Y , Su G , Cheng L , et al. Coexpression analysis identifies nuclear reprogramming barriers of somatic cell nuclear transfer embryos. Oncotarget. 2017;8(39):65847‐65859. 29029477 10.18632/oncotarget.19504 PMC5630377

[19] Zhang Z , Huang KE , Gu C , et al. Molecular subtyping of serous ovarian cancer based on multi‐omics data. Sci Rep. 2016;6:26001. 27184229 10.1038/srep26001 PMC4868982

[20] Tothill RW , Tinker AV , George J , et al. Novel molecular subtypes of serous and endometrioid ovarian cancer linked to clinical outcome. Clin Cancer Res. 2008;14(16):5198‐5208. 18698038 10.1158/1078-0432.CCR-08-0196

[21] Zhang D , Chen P , Zheng CH , Xia J . Identification of ovarian cancer subtype‐specific network modules and candidate drivers through an integrative genomics approach. Oncotarget. 2016;7(4):4298‐4309. 26735889 10.18632/oncotarget.6774 PMC4826206

[22] Zheng M , Hu Y , Gou R , et al. Integrated multi‐omics analysis of genomics, epigenomics, and transcriptomics in ovarian carcinoma. Aging (Albany NY). 2019;11(12):4198‐4215. 31257224 10.18632/aging.102047 PMC6629004

[23] Chow MT , Möller A , Smyth MJ . Inflammation and immune surveillance in cancer. Semin Cancer Biol. 2012;22(1):23‐32. 22210181 10.1016/j.semcancer.2011.12.004

[24] Geary CD , Sun JC . Memory responses of natural killer cells. Semin Immunol. 2017;31:11‐19. 28863960 10.1016/j.smim.2017.08.012 PMC5724965

[25] Ayyoub M , Pignon P , Classe JM , Odunsi K , Valmori D . CD4+ T effectors specific for the tumor antigen NY‐ESO‐1 are highly enriched at ovarian cancer sites and coexist with, but are distinct from, tumor‐associated Treg. Cancer Immunol Res. 2013;1:303‐308. 24777968 10.1158/2326-6066.CIR-13-0062-T

[26] Mahdavi M , Nassiri M , Kooshyar MM , et al. Hereditary breast cancer; Genetic penetrance and current status with BRCA. J Cell Physiol. 2019;234(5):5741‐5750. 30552672 10.1002/jcp.27464

[27] Bulanova DR , Helenius M , Sokolenko AP , Kuznetsov SG , Imyanitov EN . Response to: the GPRC5A frameshift variant c.183del is not associated with increased breast cancer risk in BRCA1 mutation carriers. Int J Cancer. 2019;144(7):1758‐1760. 30474109 10.1002/ijc.32013

[28] Fu X , Cui Y , Yang S , Xu Y , Zhang Z . MicroRNA‐613 inhibited ovarian cancer cell proliferation and invasion by regulating KRAS. Tumor Biol. 2016;37(5):6477‐6478. 10.1007/s13277-015-4507-7 26631045

[29] Jin G , Mao X , Qiao Z , Chen B , Jin F . RAP80 expression in breast cancer and its relationship with apoptosis in breast cancer cells. Onco Targets Ther. 2019;12:625‐634. 30705591 10.2147/OTT.S186981 PMC6343510

[30] Zhang J , Lin Y , Sun XJ , et al. Biomarker assessment of the CBCSG006 trial: a randomized phase III trial of cisplatin plus gemcitabine compared with paclitaxel plus gemcitabine as first‐line therapy for patients with metastatic triple‐negative breast cancer. Ann Oncol. 2018;29(8):1741‐1747. 29905759 10.1093/annonc/mdy209

[31] Sharma P , Allison JP . The future of immune checkpoint therapy. Science. 2015;348(6230):56‐61. 25838373 10.1126/science.aaa8172

[32] Raval RR , Sharabi AB , Walker AJ , Drake CG , Sharma P . Tumor immunology and cancer immunotherapy: summary of the 2013 SITC primer. J Immunother Cancer. 2014;2:14. 24883190 10.1186/2051-1426-2-14 PMC4039332

[33] Hodi FS , Chesney J , Pavlick AC , et al. Combined nivolumab and ipilimumab versus ipilimumab alone in patients with advanced melanoma: 2‐year overall survival outcomes in a multicentre, randomised, controlled, phase 2 trial. Lancet Oncol. 2016;17(11):1558‐1568. 27622997 10.1016/S1470-2045(16)30366-7 PMC5630525

[34] Cui T , Zhang L , Huang Y , et al. MNDR v2. 0: an updated resource of ncRNA‐disease associations in mammals. Nucleic Acids Res. 2018;46(D1):D371‐D374. 29106639 10.1093/nar/gkx1025 PMC5753235

[35] Liang ZY , Lai HY , Yang H , et al. Pro54DB: a database for experimentally verified sigma‐54 promoters. Bioinformatics. 2017;33(3):467‐469. 28171531 10.1093/bioinformatics/btw630

[36] Hu B , Zheng L , Long C , et al. EmExplorer: a database for exploring time activation of gene expression in mammalian embryos. Open Biol. 2019;9(6):190054. 31164042 10.1098/rsob.190054 PMC6597754

[37] Yang H , Lv H , Ding H , Chen W , Lin H . iRNA‐2OM: a sequence‐based predictor for identifying 2'‐O‐methylation sites in homo sapiens. J Comput Biol. 2018;25(11):1266‐1277. 30113871 10.1089/cmb.2018.0004

[38] Zuo Y , Li Y , Chen Y , Li G , Yan Z , Yang L . PseKRAAC: a flexible web server for generating pseudo K‐tuple reduced amino acids composition. Bioinformatics. 2017;33(1):122‐124. 27565583 10.1093/bioinformatics/btw564

