# Supplementary figures and images for: Identification of immune‐enhanced molecular subtype associated with BRCA1 mutations, immune checkpoints and clinical outcome in ovarian carcinoma
Source: J Cell Mol Med. 2020 Jan 29;24(5):2819–31. doi: 10.1111/jcmm.14830 (PMC7077593; doi:10.1111/jcmm.14830)

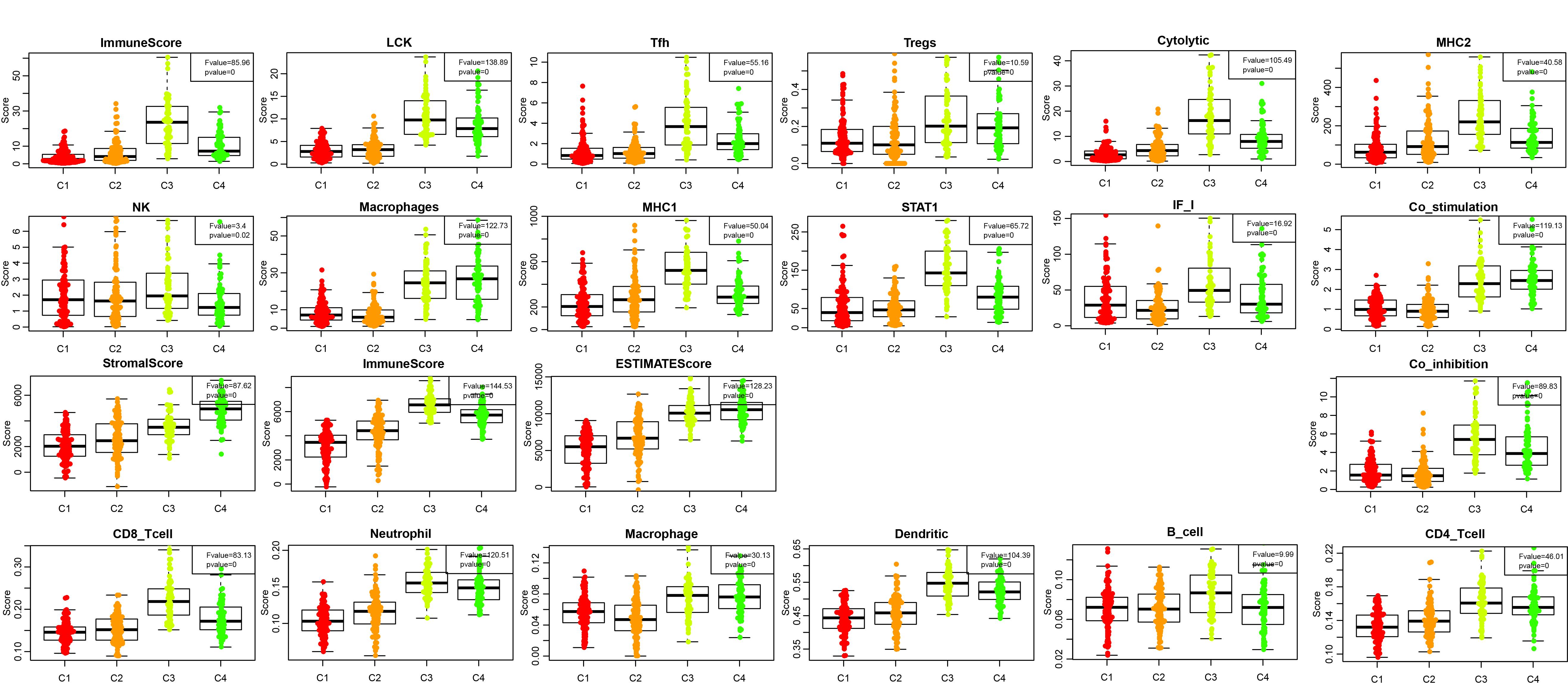

Supplement: Supplementary file 1 [file JCMM-24-2819-s001.tif]
