# Supplementary material for: Identification of immune‐enhanced molecular subtype associated with BRCA1 mutations, immune checkpoints and clinical outcome in ovarian carcinoma
Source: J Cell Mol Med. 2020 Jan 29;24(5):2819–31. doi: 10.1111/jcmm.14830 (PMC7077593; doi:10.1111/jcmm.14830)
Supplement: Supplementary file 2 [file JCMM-24-2819-s002.docx]

**STable 1 : Statistics of genes corresponding to modules**

| **Modules** | **Genes** |
| --- | --- |
| blue | 115 |
| brown | 100 |
| grey | 413 |
| turquoise | 182 |
| yellow | 61 |
